# Domestic Hazards with Neurological Significance

**Published:** 1960-03

**Authors:** A. M. G. Campbell

**Affiliations:** Neurologist, Bristol Royal Infirmary


### DOMESTIC HAZARDS WITH NEUROLOGICAL SIGNIFICANCE

BY

A. M. G. CAMPBELL, D.M., F.R.C.P.

*Neurologist, Bristol Royal Infirmary*

It is not the purpose of this paper to discuss what Dr. Boucher of the Ministry of Health has emphasized—the frequency of accidents in the home from slippery floors, hot water burns or electric fires, but to consider the more unusual domestic hazards which arise in the house or garden, with an emphasis on the neurological effects they produce. Most families have drugs of some kind in the house, which may be considered non-toxic, such as iron. It is well known that iron tablets have killed some children, for the child can mistake the pretty coloured tablet for a sweet and take an overdose of iron. It is the less obvious hazards I wish to deal with here.

*Metallic Poisonings*

In this modern age, in which new chemical compounds are frequently introduced, particularly for food preservation and cleansing, it is always possible that some new compound may be introduced which has considerable toxicity, either immediately or from its cumulative effect. About three years ago in France, a compound, to be taken orally containing organic tin, was passed as safe for the treatment of boils. It was only after nearly a hundred people had died from its effects that it was realized that the material was highly toxic, and compensation claims are still being settled. If a compound can be put on the market so easily in France, it must be realized that a similar predicament might arise in this country, before the toxic effect of a substance is realized. It took us well over a generation to associate Pink Disease in children with mercury present in teething powders. It is now generally accepted that this mercury compound was responsible for the polyneuritis and rash in this disease of children. These are but two examples of how chemical compounds, apparently innoxious, can be accepted. Heavy metals have always produced poisoning and usually give symptoms which are neurological. Organic mercury has been used as dressing for seeds and Hunter and Russell[1] described cases of poisoning from this substance which produced a specific damage to the cerebellum and the occipital lobes, producing an occipital lobe blindness.

Mercury has produced poisoning in police detectives who used mercury as finger print powder, and also in meter readers who handle the mercury in the course of their work. Symptoms produced in these cases are different from those of organic mercury as they were mainly depression, anxiety and the classical tremor. Many of them were thought to be psychological cases before it was realized that they contracted their trouble as a result of handling mercury.

Lead as a cause of domestic poisoning has been known for a long time. It is not always realized that lead can still produce poisoning n this modern age, producing a variety of symptoms such as encephalopathy with fits, coma and mental derangement, wrist drop, jaundice and colic. One of the earliest descriptions of lead poisoning was that of Sir George Baker[2] in 1767. He showed that lead poisoning was produced, in Devonshire, by the storing of cider in lead containers, and he also indicated that in Worcestershire, where wood casks were used, no poisoning occurred. Having attacked a local interest he was denounced from the pulpit for interfering with the cider industry. It is perhaps poetic justice that two years ago a case of severe lead poisoning was admitted from Devonshire to the neurosurgical unit at Frenchay. This was a woman whose symptoms were originally thought to be due to an intracerebral tumour and who had actually had a decompression for her cerebral symptoms two and a half years before. She was again admitted with signs of very high intracranial pressure, fits, mental disorder and jaundice. Mild punctate basophilia in her blood give a clue to the diagnosis and treatment with di-sodium versenate, a new chelating agent, produced a dramatic recovery of symptoms and within eight days her papilloedema had disappeared, her mentality became normal and she returned home perfectly well. After communicating with the local medical officer of health at Torrington, it was discovered that this patient lived on a farm in which pipes from a well were all lined with lead, and as the water was acid, the patient had continuously absorbed the lead for the past twelve years. Her husband and daughter were also found to have a high lead content in their blood, although they had so far suffered no ill effects. The whole water supply was altered, the lead piping being replaced by plastic pipes, at considerable cost to the local authority. The patient has remained perfectly well since this date, but it is of course possible that her daughter and husband may at a later date show symptoms when their bones decalcify. This shows that even in this country the water supply may still be contaminated with lead. Other sources of lead poisoning which have recently been described: cases of lead poisoning, a few of them fatal, occurred in the North of England when lead batteries were burned as a source of fuel in stoves for heating purposes. Lead fumes were produced and several children died. Children also get lead poisoning from licking lead paint on toys and there was a severe outbreak of lead poisoning in Queensland owing to dust from lead painted railings flaking off in a hot summer. In at least one case lead poisoning has been produced by lead paint used by actresses; poisoning from lead nipple shields and lead containers for iced lollies has been described. The lead poisoning produced by tetra-ethyl petrol produces a neurological picture but is hardly a domestic hazard.

McAlpine[3] has recently suggested that metallic poisoning may be produced indirectly by eating fish or animals that have themselves consumed a noxious compound. The discharge of factory effluents has been suggested as the cause of an obscure outbreak of poisoning in Japan, where a considerable number of fishermen and people living in the vicinity of a coastal town in Japan were affected with ataxia, blindness and motor weakness. The source of the poisoning was traced to the fish that these people ate. Crows and domestic animals also died. The first possibiity is that the effluents from a local chemical factory was discharged in a different place nearer the coast or secondly that it was due to a large amount of ammunition that had been dumped into the sea after the war, in this area, which contained a high metal content. The metals suspected originally were beryllium and selenium, neither of which has been known to produce much toxic effect on the nervous system. A recent communication from Japan appears to incriminate mercury as being the likely cause of the poisoning. The clinical signs of these cases would fit organic mercury poisoning much more easily than any of the other metals suggested.

Thallium is also a nerve poison, producing peripheral neuritis and optic atrophy, but it is not a metal which people use much and it is only used medicinally in the treatment of ringworm.

### *Insecticides*

Since the recent war there has been a larger and larger use of insecticides, both domestically and in agriculture. The commonest insecticide used is of course, D.D.T. It is not regarded as being acutely toxic and very few cases of definite poisoning in man have occurred, except by suicidal intent. It is, however, a nerve poison and acts on the insect by paralyzing the nervous system. In certain cases of poisoning in man it has produced a polyneuritis, and liver and kidney damage. It is a cumulative drug and is stored in fat. In a recent analysis of 50 women in America, taken at random, 35 were found to contain considerable quantities of D.D.T in their fat, which had accumulated merely as a result of domestic exposure to D.D.T., by using fly sprays and other compounds. We cannot tell whether this drug will have cumulative effects over a long period. It has been shown that young animals are much more susceptible to its toxic effects than adults, and after spraying pastures calves were found to be prone to epileptic fits. It is more dangerous in an oily solution used as woodworm killers because it is rapidly absorbed through the skin. These oily preparations have been known to produce polyneuritis when the person concerned has used them without due care, and absorption has taken place through the unprotected skin. Polyneuritis has resulted and this may be due either to D.D.T. or to pentachlorophenol which also has been found to produce polyneuritis in workers who make the compounds[4].

More toxic insecticides are used in agriculture. Organic mercurial derivatives have already been mentioned; the dinitro-orthocresol derivatives are highly toxic, usually producing poisoning in people who use the sprays without due precaution, producing rapid increase in basal metabolic rate, rapid pulse rate, sweating and collapse.

The organic phosphorous compounds are also highly poisonous producing paralysis at the neuromuscular junction. Luckily, however, atropine is an effective antidote. If the crops are gathered too soon after spraying, say anything less than three weeks after exposure, particularly in a dry summer, there is a risk that the vegetables will contain enough contamination to affect those who eat them. There was some anxiety during the last dry spell that not enough rain had fallen to wash away the toxic effects of such compounds. It is probable that symptoms of poisoning which occurred in Essex about two or three years ago were a result of contamination with organic phosphorous sprays, although luckily there were no fatalities. The great toxicity of insecticides was emphasized recently by the contamination of a railway waggon in Wales as a result of diendrine having leaked from a drum: this waggon was subsequently used to carry flour to a town in Wales. Flour was then baked into bread and distributed throughout the town and within a few hours of eating the bread over one hundred people were ill with coma, epileptic fits, drowsiness and vomiting. The contamination of the flour was extremely small which showed the excessive toxicity of this substance. Luckily there were no fatalities.

*Other Poisons*

While on the subject of flour, ergot poisoning of bread as a result of impure flour and its contamination by a toxic fungus produced several deaths in a French town with severe mental illness, about three years ago. No cases of ergot poisoning have occurred from this source in this country for the last twenty years.

Mellanby[5] has found that cases of epileptic fits in dogs were definitely associated with the eating of white bread which had been bleached by the addition of agene and it was thought that epilepsy might be produced in human beings in a similar way. All experiments, however, have failed to incriminate agenized flour as producing epilepsy in man or in other animals, and it would seem that the dog is peculiarly susceptible to this compound.

Before leaving organic compounds, mention should be made of carbon tetrachloride. This poison can produce some damage to the nervous system, but usually produces severe renal and liver damage. It is present, of course, in many proprietary compounds used in cleaning clothes and it is only dangerous if used in an enclosed space where the persons may absorb the fumes. For example two naval ratings working in a submarine, were poisoned as a result of cleaning their uniforms in an enclosed space without adequate ventilation.

The importance of the toxicity of the orthocresol derivatives has been emphasized recently once again. Several outbreaks of motor polyneuritis have followed the accidental contamination of food by these highly toxic derivatives. Ginger wine became contaminated in the United States, margarine in Germany after the last war and a few cases of poisoning also occurred in Liverpool. Recently, however, a major outbreak in Morocco has occurred where it is estimated that probably 10,000 people were poisoned by the contamination of olive oil, used in cooking, by surplus jet fuel oil which was mixed with the vegetable oil and sold on the market by some ingenious Arab profiteers. The epidemic reached such proportions that the Moroccan Government asked for help from the World Health Organization and it became clear after investigation that only certain Arabs, using the poorer qualities of cooking oil, had become affected by the oil which appears to have a selective effect on the motor nerve, and to a lesser degree on the spinal cord. Luckily most cases recovered. Any outbreak of multiple paralysis of this kind should be suspected of being due to a poison, possibly orthocresol.

An interesting epidemic of epilepsy in babies in the United States was found to be due to the fact that a certain proprietary dried milk had been overheated with the result that the essential vitamin, pyrodoxine, had been destroyed; and as pyrodoxine is necessary for the development of the nervous system, epilepsy had resulted in these babies fed on this compound, due to a deficiency syndrome.

Whether we shall suffer any ill effects from the use of detergents, which is the other major change in domestic practice, no-one can say. All I know is that I heard of an M.O.H. who, when he washed up, always insisted that the china was washed in hot water after the use of detergents. He himself, however, has recently died of a carcinoma of the bronchus, possibly related to the fact that he was a heavy smoker. Detergents do not appear to be toxic to animals, except when they are put directly into the spinal fluid.

*Infections from Pets*

Having spent some time on chemicals I would now like to turn to pets in the home. There seems to be a myth in the lay minds that animal diseases are not transferable to man. One of the most serious groups of disease that has been transmitted to many by animals is due to leptospiral infections. A common variety is contracted from rats, either by bathing in water which is rat-infested or through abrasions caused by working in conditions where rats abound. Cases have occurred in Bristol from swimming in local streams and even from swimming in the Henleaze swimming pool. Certain industrial workers have contracted the disease, particularly fish workers in Aberdeen and miners in South Wales. The most unusual case which occurred was an epileptic who fell, after a fit, into a small pool of water and absorbed the infected organisms as a result of being immersed in water which must have previously had rat contacts. Another variety of leptospirosis is carried by dogs and about 20 per cent of the canine population of a large city appear to be infected[6]. The dog infection is also contracted by bathing and one patient acquired it through bathing in a pool in which a dead dog was later found. A commoner source of infection is by contamination of the skin with dog's urine which contains the organisms. A husband and wife living in Bristol both contracted a meningitis by this method of infection. Both types are commoner in the summer months, probably due to bathing.

Infection of human beings with psittacosis from budgerigars and parrots is not as common as it was, but cases producing an atypical pneumonia still crop up from time to time. Recently there has been some controversy over whether budgerigars carry poliomyelitis. It has always been suggested that poliomyelitis virus has an animal reservoir which may possibly spread the disease from place to place and the finding of a virus in a budgerigar was an interesting isolated observation which has not been confirmed.

Equine encephalitis is a disease which is confined to the United States of America but undoubtedly in this condition the virus is spread by birds for many miles and the bird does not apparently suffer any ill effects.

Dogs have been proved to be carriers of human tuberculosis, and Swedish workers have shown quite clearly that one dog infected six members of a family with tuberculosis, its original master died from the disease and the dog was looked after by another family who immediately contracted tuberculosis.

I have been interested in the possibility of cats being associated with polyneuritis of an infective type[7] and the scratch of a cat produces cat scratch fever with enlargement of the glands and skin lesions. Mice are also responsible for lymphocytic meningitis where food has been contaminated by mice.

A protozoal disease of animals, known as toxoplasmosis, has also been shown to infect man, and the disease has many symptoms including cerebral complications, eye lesions and glandular enlargement. It is probable that this disease is very prevalent in the animal kingdom, particularly in dogs, cats, rabbits and even in birds. From positive agglutination and complement fixation tests done on the population of cities, it is estimated that between 30–50 per cent of people are infected at some time in their lives without its producing any serious symptoms, but about one in five hundred people may get serious trouble. It is obvious, therefore, from these facts that children should not allow animals to lick their faces, that care should be taken not to allow animal excretion to contaminate open sores or utensils and that plates used by dogs should not be used by humans without adequate sterilization.

*Neuritis from Compression*

The housewife during the course of her duties is particularly prone to suffer from neuritis, owing to the irritation of peripheral nerves. It was pointed out by Walsh[8] that neuritis occurred in middle aged women mainly owing to repeated domestic duties involving lifting, washing, polishing, vacuum cleaning, etc., and he suggested that the tone of the shoulder group of muscles deteriorates. When the shoulder drops there is too little room for the outlet for the nerves forming the brachial plexus and as a result, particularly at night, these nerves are irritated and the patient gets painful neuritis down the arm. It is notorious that these symptoms are worse on waking, and pass off during the day; they are directly related to the amount of work done during the previous day, and the patient tends to lose his symptoms on holiday. Concentration, however, on this shoulder girdle syndrome had led to the neglect of the importance of wrist compression of the median and ulnar nerves, but in particular the median nerve, where it lies in the carpal tunnel and where particular movements of flexion with the wrist in hyper-extension, such as the classical example of hand milking, is apt to produce compression and irritation of the median nerve in the carpal tunnel. The carpal tunnel syndrome of median weakness and irritation is now common as a cause of pain in the arm and particularly the hand, and a great deal has been done by operations to relieve these sufferers. The diagnosis can usually be established by the typical median distribution of the sensory loss and the wasting of the appropriate thenar eminence. The superficial branch of the ulnar nerve can also be compressed at the wrist producing a motor weakness and often no sensory loss, and the ulnar nerve can also be compressed as a result of leaning on the elbows, such as the medical student indulging in excessive study, particularly if the nerve lies superficially in the groove at the back of the elbow. It is important if possible, to find out the particular pressure or movements which produced irritation of these nerves and one patient I have seen undoubtedly produced his ulnar paralysis by his method of sleep. He slept with his arm tucked underneath his head producing an ulnar nerve compression. This was so firm a conditioned reflex that his arm had to be put in plaster to prevent his adopting this posture unknowingly. As a result of this treatment his ulnar nerve paralysis cleared up completely. People are sometimes apt to get compression of the radial nerve from the use of crutches and a more unusual method of compression is from another human body compressing the arm against a hard surface, such as the back of a cinema seat or the ground. This results in a drop wrist, which can be somewhat embarrassing to both parties; the compression may be as short as two hours and the recovery as long as six weeks. There are other forms of pressure neuritis which I need not deal with and are less common, but a careful history of what the patient does is essential to establish the correct diagnosis and appropriate prevention and treatment.

In conclusion, I have tried to link together various forms of possible neurological hazard which are very diverse in their aetiology, but the majority of them occur in the home and may be of some interest to stimulate observation of other conditions of a similar nature. It is of interest that a large number of the conditions I have described have only been recognized within the last twenty years.

### REFERENCES

[1] Hunter, D. and Russell, D. S. (1954). *J. Neurol. Psychiat.*, **17**, 235.
[2] Baker, *Med. Tr. Coll. Phy.* (London, 1772), **1**, pp. 175.
[3] McAlpine, D. (1959). Personal Communication.
[4] Campbell, A. M. G. (1949). *Lancet*, **2**, 1178.
[5] Mellanby, E. (1931). *Brain*, **54**, 247.
[6] Campbell, A. M. G., Macrae, J., Manderson, W. G., Sumner, K. C. and Broom, J. C. (1950). *Brit. Med. J.*, **1**, 336.
[7] Campbell, A. M. G. (1958). *Proc. R. Soc. Med.*, **51**, 157.
[8] Walshe, F. M. R., Jackson, H. and Wyburn-Mason, R. (1945). *Brain*, **67**, 141.

